# Metastatic Oropharyngeal Squamous Cell Carcinoma Presenting as an ST-Elevation Myocardial Infarction (STEMI) in a 55-Year-Old Male

**DOI:** 10.7759/cureus.76691

**Published:** 2024-12-31

**Authors:** Abiram Sivanandam, Divya Viswanathan, Anand Shah, Rafael T Manotas, Vincent Yeung

**Affiliations:** 1 Internal Medicine, Rutgers University New Jersey Medical School, Newark, USA; 2 Hematology and Oncology, Rutgers Cancer Institute of New Jersey, New Brunswick, USA

**Keywords:** metastatic squamous cell carcinoma, pericardial effusion, pericardial metastases, squamous cell neoplasm, st elevations

## Abstract

A 55-year-old Hispanic male was admitted to the medicine service for tachycardia and shortness of breath of onset three to four days prior to arrival. His prior medical history was notable for Stage IVa oral squamous cell carcinoma (OSCC) with post subtotal glossectomy, floor of the mouth resection with segmental mandibulectomy, bilateral selective neck dissection (SND) of cervical levels I-IV, and chemoradiation therapy. His post-treatment positron emission tomography (PET) showed no metastatic disease. His electrocardiogram (ECG) was notable for ST elevations in leads III, aVF, V3, and V4 concerning for an inferior wall ST-elevation myocardial infarction (STEMI) requiring emergent cardiac catheterization, which ultimately revealed no coronary obstruction. A CT angiogram of the chest was obtained to rule out pulmonary embolism but incidentally revealed a new pericardial and left pleural effusion, as well as an enlarged left supraclavicular lymph node concerning for possible metastatic disease. A transthoracic echocardiogram revealed a mass-like structure in the pericardium. A biopsy of the pericardium and lymph node revealed metastatic SCC. Due to negative cardiac catheterization findings, the patient's ECG changes were thought to be secondary to metastatic disease. Head and neck cancer rarely metastasizes to the heart. The onset of new chest pain or shortness of breath in the setting of malignancy should be carefully evaluated for ACS but also merits evaluation for metastatic disease as evidenced by this patient’s clinical presentation.

## Introduction

Head and neck cancers are squamous cell carcinomas arising from the oral cavity, paranasal sinuses, pharynx, and larynx. Oral squamous cell carcinomas (OSCCs) arise from the mucosal surface of the oral cavity and oropharynx and account for 90% of all oral malignancies, with the most common location being the tongue and floor of the mouth [1-3]. They are debilitating malignancies with clinical features and treatment effects having a significant negative impact on the quality of life, affecting appearance, speech, swallowing, and psychosocial well-being [1].

Smoking and alcohol consumption are the most common risk factors associated with OSCC [4]. Human papillomavirus (HPV) infection has been previously associated with an increasing incidence of cancers arising from the oropharynx, albeit with an improved prognosis as compared to OSCC not associated with HPV [5]. The incidence of HPV and the oral cancers associated with its infection have been observed to be increasing in recent years [6]; however, they have higher survival rates than those with OSCC not associated with HPV [6].

Clinical features vary according to the stage and primary site of the tumor. Tumors of the oral cavity present as nonhealing ulcers, masses, ill-fitting dentures, or painful lesions. OSCC can have regional and distant metastasis, with the most common site for OSCC metastasis being cervical lymph nodes. More than half of oral cancers are at an advanced stage (stages 3 and 4) on diagnosis, making it likely to have a poor prognosis once diagnosed due to a higher likelihood of presenting with metastatic disease [7]. In the early stages, it characteristically metastasizes to the regional lymph nodes through draining lymphatics, which is usually considered the first indication for spread [8]. Metastases in OSCC often begin with local invasion and dissemination through lymphatic and hematogenous spread [8]. Patients diagnosed at an advanced stage of the disease are more likely to present with visceral metastases [8]. Among patients with distant metastasis, the usual sites are lung, mediastinum, liver, bone, and skin [9].

Most cardiac malignancies are typically secondary in nature rather than a primary process, as primary cardiac tumors are rare [10,11]. The most common cardiac metastases are neoplasms of the lung, breast, lymphoma, skin, and melanoma, with head and neck squamous cell cancers being a rare cause of metastasis.

The mechanism by which tumors metastasize to the heart is unclear but may include similar lymphatic and hematogenous pathways seen in other sites of OSCC metastases [9]. The most common site of the heart affected by metastatic disease is the pericardium [10]. The reported incidence of cardiac metastasis from autopsies of cancer patients is highly variable in the literature with an average incidence of 4.71% from any type of primary malignancy [10]. However, this number may be under-reported, as most metastases to the heart are clinically silent and are often not detectable. In 1955, Gassman et al. studied 4,124 autopsies from a Veterans Affairs hospital in Illinois of which 126 cases of primary tongue carcinoma were biopsied [12]. Among these 126 cases, only 2 patients presented with metastases to the heart and pericardium (1.5%). A more recent study by Manojlović reported that 9 of 39 patients who were autopsied were found to have cardiac metastases and were diagnosed with OSCC [13]. The limited sample size of these postmortem studies highlights the need for further research on this presentation. 

## Case presentation

We present the case of a 55-year-old Hispanic male with a prior medical history that was notable for Stage IVa OSCC. He presented from his oncologist's office for tachycardia with a heart rate in the 130s and worsening shortness of breath of onset 3-4 days prior to arrival in the emergency department.

The patient's OSCC was previously treated with subtotal glossectomy, floor-of-the-mouth resection with segmental mandibulectomy, bilateral selective neck dissection of cervical levels I-IV, and chemoradiation therapy with weekly cisplatin. His post-treatment PET scan showed no metastatic disease eight months prior to his presentation.

**Figure 1:**
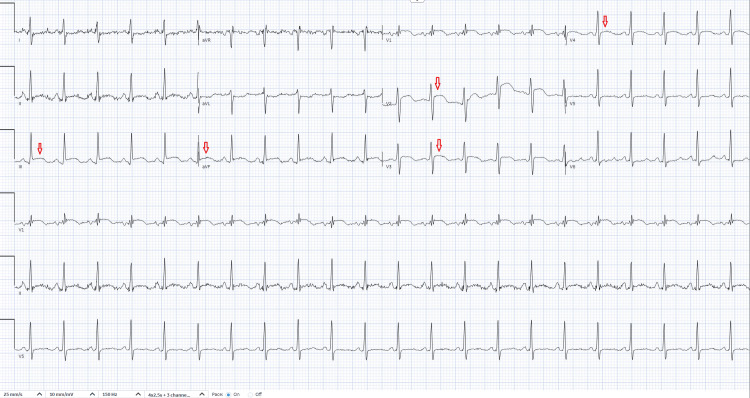
Initial ECG obtained ST elevations are noted in leads III, aVF, V2, V3, and V4 as denoted by the red arrows.

**Figure 2:**
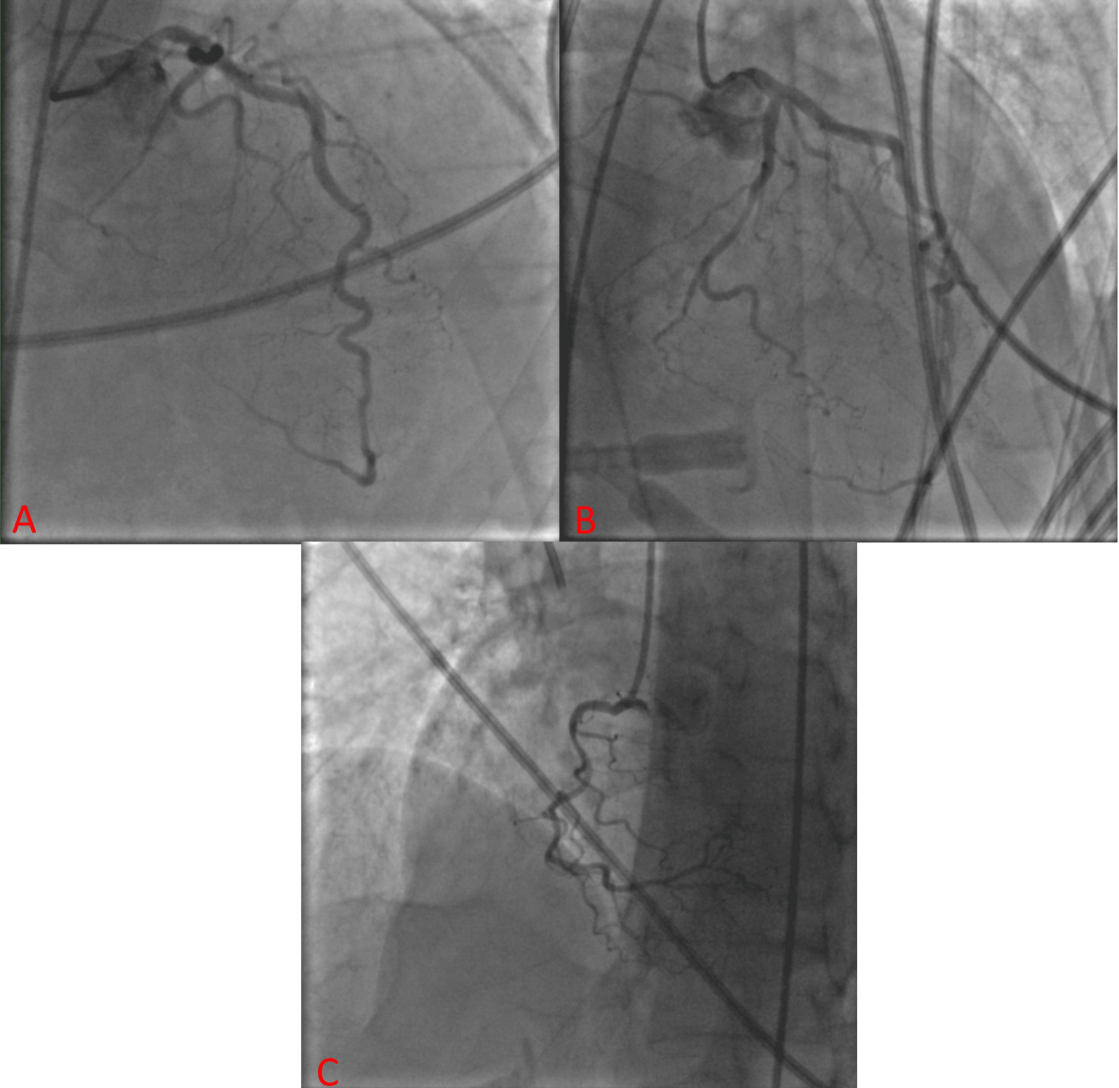
Left heart catheterization Visualization of the left anterior descending (LAD) artery (A), left circumflex (LCx) artery (B), and right coronary artery (RCA) (C) shows no significant obstructive stenosis.

The patient’s initial electrocardiogram (ECG) showed ST elevations in leads III, aVF, V2, V3, and V4 (Figure 1) concerning for acute coronary syndrome (ACS). The patient underwent emergent cardiac catheterization, which did not reveal any coronary obstruction (Figure 2).

**Figure 3:**
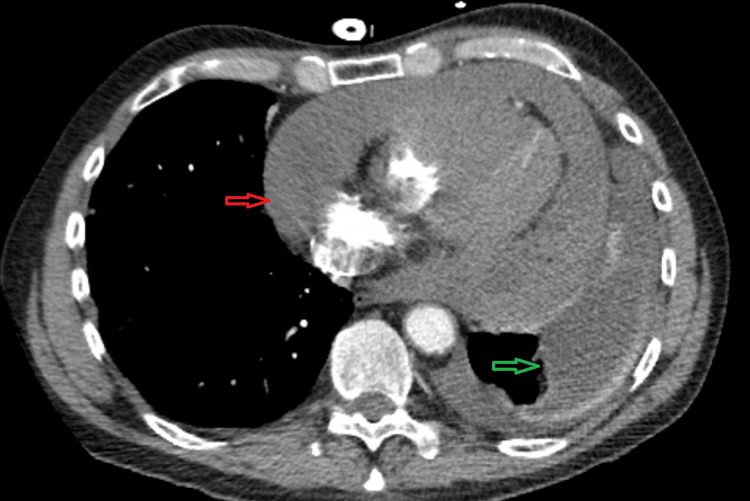
CT angiogram of the chest A large pericardial effusion (red arrow) and left-sided pleural effusion (green arrow) are observed.

A computerized tomography (CT) angiogram of the chest (Figure 3) showed a large pericardial effusion with new mediastinal lymph nodes and a large left pleural effusion but no pulmonary embolism. A pericardial window and drain were placed by cardiothoracic surgery (CTS) and interventional radiology (IR), respectively, and the patient was initiated on colchicine without improvement of his symptoms. A thoracentesis was performed, and the pathology of the fluid was consistent with metastatic squamous cell carcinoma (SCC).

Due to continued symptoms of chest pain and shortness of breath, a mutual decision was made between the family and Oncology to start chemotherapy with one cycle of carboplatin and 5-fluorouracil (5-FU) to obtain disease control. Following treatment, the patient was found to be persistently tachycardic, hypotensive, and unresponsive to IV fluid boluses. A repeat ECG showed persistent ST elevations in V2-V4 with a bedside echocardiogram showing a stable pericardial effusion without evidence of right heart strain.

The patient was taken for a repeat cardiac catheterization and no coronary obstruction was observed. Ultimately, the patient requested to stop treatment with chemotherapy given his continued and worsening symptoms. After a goals-of-care discussion between oncology, palliative care, and the patient's family, the patient opted for hospice care given the severity of his metastatic disease without the resolution of symptoms.

## Discussion

Cardiac metastases in patients with cancer are difficult to diagnose antemortem and are often discovered incidentally or once patients are symptomatic, leading to further evaluation [14]. In our patient’s case, symptoms of tachycardia and shortness of breath led to assessment for potential ACS, as well as concern for pulmonary embolism given the patient's hypercoagulable state due to his cancer history, but lower initial suspicion for metastatic involvement of the heart.

We have identified five other cases of primary OSCC with metastases to the heart that presented with ST elevations on EKG [15-19]. To the best of our knowledge, only one other case of SCC with cardiac metastases has presented with acute concern for ACS, leading to emergent cardiac catheterization [19]. In this case, the patient presented with mild ST elevations in V4 and V5. Cardiac catheterization was performed on this patient and showed no obstructive disease and myocardial infarction was ruled out due to a lack of rise in cardiac enzymes. Although this patient underwent a course of cisplatin, 5-FU, bleomycin, and methotrexate, he ultimately died from multiorgan failure related to metastatic squamous cell carcinoma. Our patient presented similarly, with mild ST elevations in the anterolateral leads concerning for ACS and pericardial effusion, without evidence of obstructive disease on coronary angiography. Our patient had a similar time course following diagnosis and treatment of metastatic OSCC with cardiac involvement, highlighting the poor prognosis of metastatic disease to the heart. In cases where catheterization was deferred, the patients did not have significant risk factors warranting invasive intervention, and metastatic disease was visualized on echocardiography or cardiac imaging.

Treatments for cardiac metastases have been shown to be focused on symptom management with drainage of pericardial fluid if present and palliative chemotherapy and radiation [14]. In patients presenting with a cardiac mass, surgical resection has not proven to be successful [14]. The five-year survival rate of patients diagnosed with OSCC without metastatic disease has been shown to be 90% [20], decreasing to 25-40% if presenting with lymphatic metastases. Patients with cardiac metastases were found to have an average survival of 3.5 months [20], much poorer than those with regional lymphatic spread.

Due to the rare nature of cardiac metastases from primary OSCC, screening with cardiac imaging is not routine. However, there may be value in using an echocardiogram as a tool for early evaluation in patients with head and neck cancers for potential cardiac involvement when patients report atypical cardiac symptoms. The use of transthoracic echocardiogram (TTE), cardiac CT (CCT), and cardiac magnetic resonance imaging (CMR) appear to be the most common imaging modalities to view cardiac metastases. TTE provides the most rapid and cost-effective evaluation of the heart for pericardial effusion, large masses, thrombi, and hemorrhage [21]. However, patient habitus, field of view, and evaluator performance may limit diagnostic value. CCT has been shown to have high sensitivity for pericardial masses, as well as calcification [21], proving a useful tool in the diagnosis of patients with suspected cardiac involvement considering speed and cost. CMR has been described to have diagnostic value for suspected cardiac metastases with myocardial involvement [21]. However, CMR is limited by time, cost, and availability. Utilizing appropriate diagnostic imaging may be useful in developing a methodology in which cardiac metastases may be readily identified.

## Conclusions

Metastatic disease to the heart from any primary malignancy is uncommon and presents variably. The incidence of OSCC metastases to the heart is even less likely, with only a handful of prior known cases. The differential diagnosis of these patients must remain broad, as life-threatening etiologies such as ACS, pulmonary embolism, and infection must be ruled out initially. Metastatic involvement of the heart should be considered in patients with known cancer history and new-onset cardiac symptoms. There is merit in more extensive cardiac evaluation in these patients, as it is difficult to differentiate the etiology of these symptoms. The comprehensive use of cardiac biomarkers, EKG and echocardiographic evaluation, radiographic imaging, and clinical evaluation of the patient can help narrow the diagnosis in patients with such presentations. In our patient’s case, cardiac catheterization was ultimately chosen, as there was no obvious explanation for the initial EKG findings. Further research is warranted to help refine a methodology such as a risk stratification tool or standardized imaging that may help differentiate ACS from cardiac metastases in this population.

## References

[1] Valdez JA, Brennan MT (2018). Impact of oral cancer on quality of life. Dent Clin North Am.

[2] Tan Y, Wang Z, Xu M (2023). Oral squamous cell carcinomas: state of the field and emerging directions. Int J Oral Sci.

[3] Noguti J, Gomes De Moura CF, De Jesus GPP, Pereira Da Silva VH, Hossaka TA, Fujiyama Oshima CT, Ribeiro DA (2012). Impact of oral cancer on quality of life. Cancer Genomics & Proteomics.

[4] Wang X, Xu J, Wang L, Liu C, Wang H (2015). The role of cigarette smoking and alcohol consumption in the differentiation of oral squamous cell carcinoma for the males in China. J Cancer Res Ther.

[5] Gillison ML, Koch WM, Capone RB (2000). Evidence for a causal association between human papillomavirus and a subset of head and neck cancers. J Natl Cancer Inst.

[6] Gribb JP, Wheelock JH, Park ES (2023). Human papilloma virus (HPV) and the current state of oropharyngeal cancer prevention and treatment. Dela J Public Health.

[7] Abati S, Bramati C, Bondi S, Lissoni A, Trimarchi M (2020). Oral cancer and precancer: a narrative review on the relevance of early diagnosis. Int J Environ Res Public Health.

[8] Tímár J, Csuka O, Remenár E, Répássy G, Kásler M (2005). Progression of head and neck squamous cell cancer. Cancer Metastasis Rev.

[9] Kotwall C, Sako K, Razack MS, Uma Rao, Bakamjian V, Shedd DP (1987). Metastatic patterns in squamous cell cancer of the head and neck. Am J Surg.

[10] Nova-Camacho LM, Gomez-Dorronsoro M, Guarch R, Cordoba A, Cevallos MI, Panizo-Santos A (2023). Cardiac metastasis from solid cancers: a 35-year single-center autopsy study. Arch Pathol Lab Med.

[11] Bussani R, De-Giorgio F, Abbate A, Silvestri F (2007). Cardiac metastases. J Clin Pathol.

[12] Gassman HS, Meadows R, Baker LA (1955). Metastatic tumors of the heart. Am J Med.

[13] Manojlović S (1990). Metastatic carcinomas involving the heart. Review of postmortem examination. Zentralbl Allg Pathol.

[14] Shafiq A, Samad F, Roberts E, Levin J, Nawaz U, Tajik AJ (2019). Squamous cell carcinoma of the tongue with metastasis to myocardium: report of a case and literature review. Case Rep Cardiol.

[15] Chen J, Craft C, Panakos AW, Marhefka GD (2016). Squamous cell carcinoma metastatic to the heart mimicking ST-elevation myocardial infarction. Med Forum.

[16] Hsu JY, Lin HY, Yang YP (2022). Oral squamous cell carcinoma metastasizing to the heart: a case report from Taiwan. Cancer Manag Res.

[17] Tandon V, Kethireddy N, Balakumaran K, Kim AS (2019). Metastatic squamous cell carcinoma to the heart: an unusual cause of ST elevation—a case report. Eur Heart J Case Rep.

[18] Werbel GB, Skom JH, Mehlman D, Michaelis LL (1985). Metastatic squamous cell carcinoma to the heart. Unusual cause of angina decubitus and cardiac murmur. Chest.

[19] Rivkin A, Meara JG, Li KK, Potter C, Wenokur R (1999). Squamous cell metastasis from the tongue to the myocardium presenting as pericardial effusion. Otolaryngol Head Neck Surg.

[20] Kerndt CC, Nessel TA, Bills JA, Shareef ZJ, Balinski AM, Mistry DT (2021). Systematic review: cardiac metastasis of lingual squamous cell carcinoma. Spartan Med Res J.

[21] Lichtenberger JP 3rd, Reynolds DA, Keung J, Keung E, Carter BW (2016). Metastasis to the heart: a radiologic approach to diagnosis with pathologic correlation. AJR Am J Roentgenol.

